# Isolated Chorioretinal Coloboma: A Case Report

**DOI:** 10.7759/cureus.28048

**Published:** 2022-08-16

**Authors:** Taimoor A Khan, Talha Liaqat, Muhammad Shahid, Teyyeb A Janjua, Abdul Rauf

**Affiliations:** 1 Ophthalmology, Armed Forces Institute of Ophthalmology, Rawalpindi, PAK; 2 Medicine, Army Medical College, National University of Medical Sciences (NUMS), Rawalpindi, PAK

**Keywords:** retinal detachment, retinopexy, congenital abnormalities, visual field defect, chorioretinal coloboma

## Abstract

Ocular coloboma is a rare congenital anomaly that arises due to an abnormality in embryogenesis. It occurs due to failed fusion of the embryonic fissure resulting in a persistent defect. Colobomas may present with vision loss but are most commonly asymptomatic and diagnosed incidentally. In this article, we present a case of asymptomatic chorioretinal coloboma diagnosed on routine screening. The patient was managed with prophylactic argon laser retinopexy to prevent complications leading to visual impairment in the future.

## Introduction

Coloboma is derived from the Greek word "koloboma," which means mutilated or curtailed [1]. Ocular coloboma is a condition caused by the failure of closure of the embryonal fissure during embryogenesis [2]. Coloboma itself is a general term that can be used to describe any defect present within the ocular structures. Depending on the involved structure, colobomas can be said to be either typical or atypical. Colobomas involving the inferior/inferonasal part of the fundus are said to be typical, while colobomas occurring elsewhere in the eye are said to be atypical. The association of failure of closure of embryonal fissure is most strongly associated with typical colobomas [2]. Colobomas can involve any ocular structure including the iris, the ciliary body, the choroid, and the retina. Colobomas are rare genetic defects that are associated most commonly with vision loss. They can occur in isolation or as a part of genetic disorders such as CHARGE syndrome (coloboma, heart defects, choanal atresia, retarded growth and development, genital abnormalities, and ear anomalies) [3]. Patients with chorioretinal colobomas are at risk for the development of retinal detachment and chorioretinal neovascularization [4]. According to the literature, the occurrence of chorioretinal colobomas is 0.14% of the general population with 40% of these experiencing retinal detachment at some point in their lives [5]. Here, we present the case of a 26-year-old male who presented with isolated chorioretinal inferonasal coloboma.

## Case presentation

**Figure 1:**
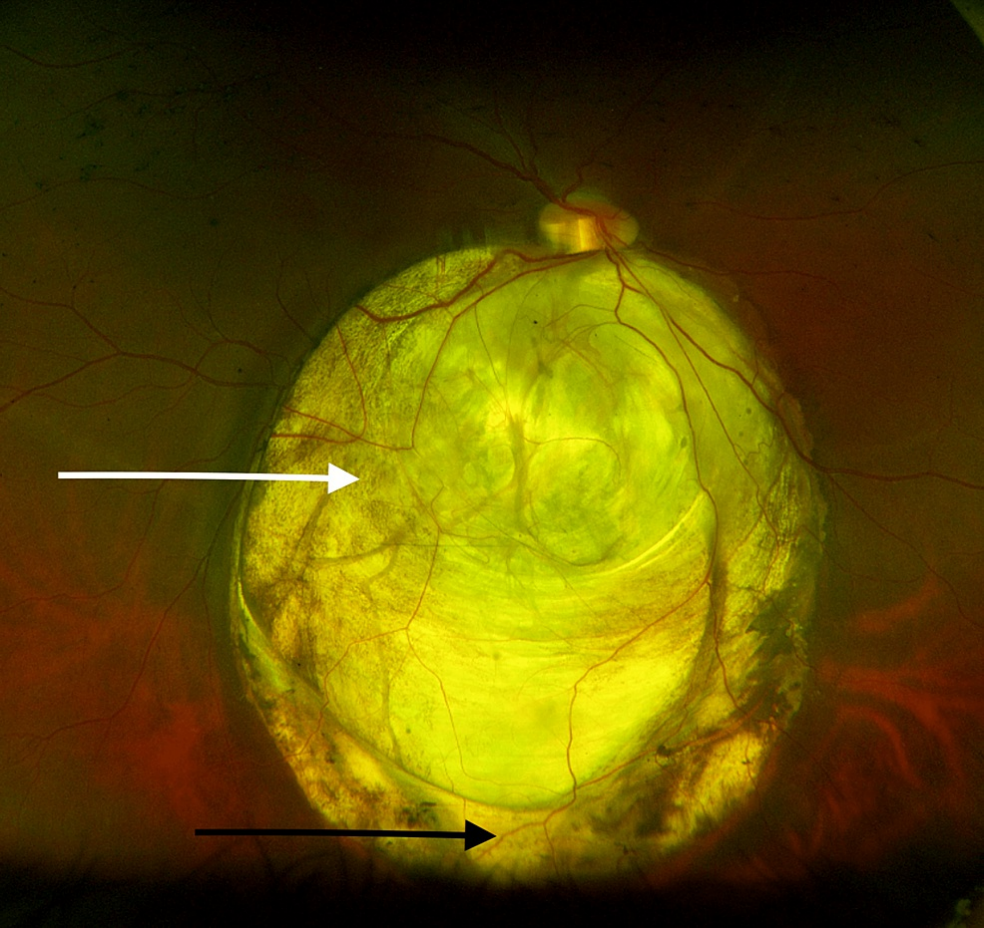
Isolated inferonasal chorioretinal coloboma image Isolated inferonasal chorioretinal coloboma 10 × 13 mm pushing the optic disc superiorly (white arrow) with an area of abnormal vitreoretinal traction (black arrow)

A 26-year-old Caucasian male from Rawalpindi, Pakistan, reported to our ophthalmic clinic for pre-employment ophthalmic screening. He had no visual complaints. On examination, his bilateral unaided distance visual acuity was 6/6. The anterior segment examination in both eyes was unremarkable. The pupils were round, regular, and equally reactive to light and phakic lens. His dilated posterior segment examination of the right eye revealed a large isolated inferonasal chorioretinal coloboma approximately 10 × 13 mm in size pushing the optic disc superiorly with the edges of the coloboma, giving the impression of abnormal vitreoretinal traction, as seen in Figure 1.

**Figure 2:**
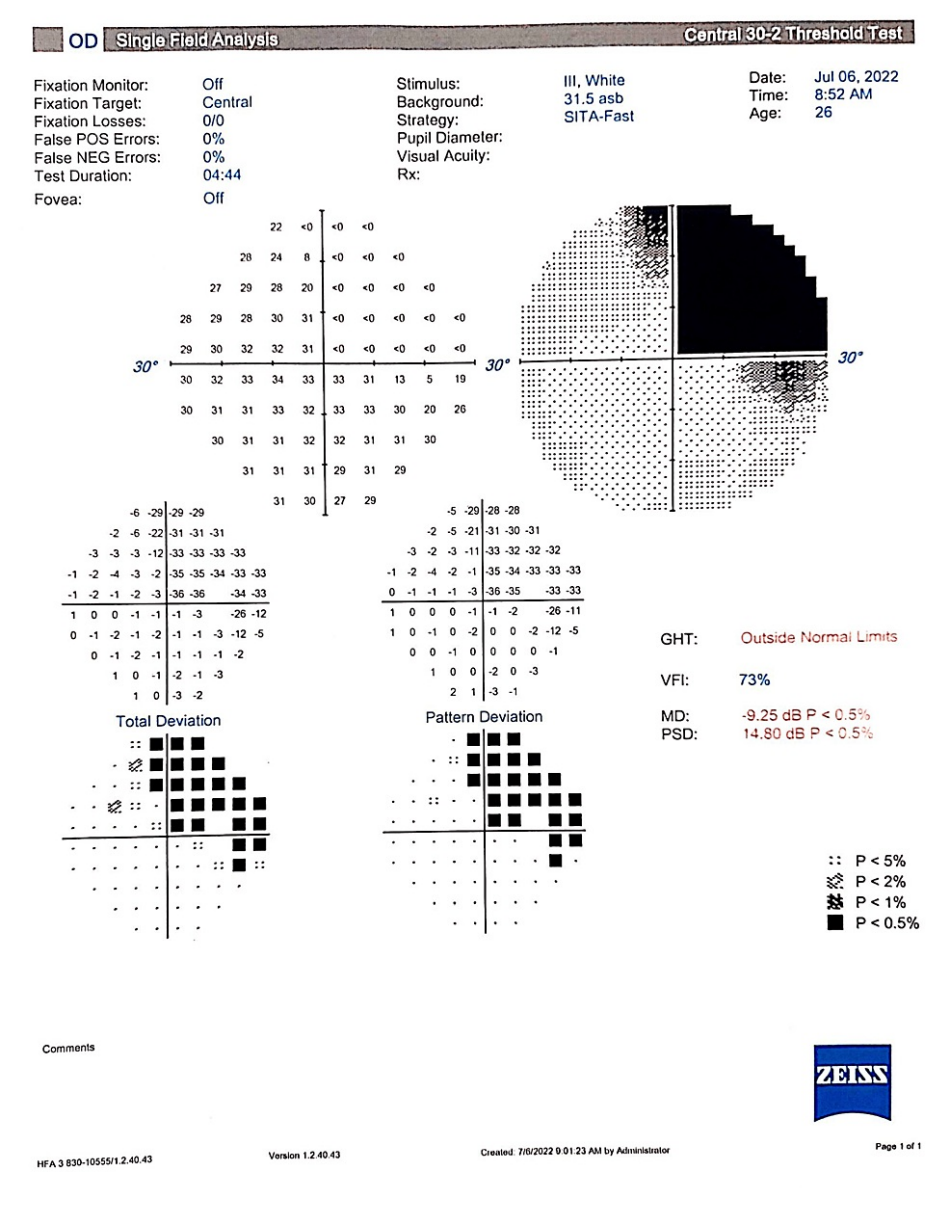
Visual field 30-2 with SITA Fast protocol showing a superotemporal field defect in the right eye SITA: Swedish interactive thresholding algorithm

The intraocular pressure (IOP) of both eyes was 15 mmHg. His visual field 30-2 with the Swedish interactive thresholding algorithm (SITA) Fast protocol revealed a superotemporal field defect in the right eye corresponding to the colobomatous defect shown in Figure 2.

We ordered Optos ultra-widefield imaging for the pictorial record, and a 360-degree prophylactic argon laser retinopexy (pattern scan laser photocoagulator {PASCAL}) was performed around the coloboma to prevent a retinal tear/retinal detachment in the future. Following the procedure, the patient was counseled regarding potential complications and kept on a six-month follow-up.

## Discussion

Ocular coloboma is a rare condition. Different studies have been conducted to find its occurrence. Population-based studies have estimated the prevalence rates for colobomas to range from 3.7/100,000 from a Hungarian national registry to 8/100,000 in Scotland [3]. The normal development of the human eye involves the formation of multiple embryonic structures. One of these structures is the optic fissure. By the fifth week of gestation, the two margins of the optic fissure displace the periocular mesenchyme to fuse in the central region of the fissure. Abnormal closure of this fissure is what leads to the defect known as coloboma [6].

Most cases of coloboma present as asymptomatic individuals diagnosed on routine screening [6]. Our patient was also asymptomatic, and a pre-employment screening revealed the defect. While it can occur as an incidental diagnosis in many cases, the presentation can vary depending on the patient. It may present as an abnormality noticed by parents/physicians because of an anomalous-looking eye. Colobomas can also cause chronically reduced vision even if the retinal detachment is not yet present. Patients can also present with acute vision reduction due to retinal detachment. Another presentation of clinically apparent coloboma is leukocoria. Large colobomas can cause a white pupillary light reflex [2].

Certain factors have been associated with an increased risk of colobomas. Environmental factors associated with increased coloboma risk include vitamin A deficiency, thalidomide exposure, and fetal alcohol syndrome [6]. None of such associations were found in our patient. Other risk factors such as increasing paternal age have also been associated with an increased risk of coloboma development [3]. The underlying genetic cause of colobomas has been difficult to identify. In the case of syndromic colobomas such as CHARGE syndrome, Treacher Collins syndrome, and Patau syndrome, the underlying genetic defect is often found, but in cases of non-syndromic colobomas, more than 70% of individuals do not have identified underlying genetic defect [3,6].

The most important complication of chorioretinal colobomas is retinal detachment with a prevalence ranging from 2.4% to 47.5% [2]. Prophylactic laser coagulation is protective and reduces the development of rhegmatogenous retinal detachment (RRD). According to one study, the likelihood of developing RRD was 10.606 times more in those individuals who did not receive prophylactic laser treatment as compared with those who received prophylactic laser coagulation [7].

The management of retinal detachment depends on the individual case, and its treatment has evolved over time. The mainstay of treatment is retinopexy. Multiple approaches are available to perform retinopexy. Cryopexy and retinopexy are both found to be equally effective. In our case, argon laser retinopexy was done. Retinal detachment may occur even after retinopexy [8]; thus, we kept our patient on a six-month follow-up to augment retinopexy if required. Danger signs of RRD were also explained in a detailed counseling session.

## Conclusions

Colobomas are a rare ocular pathology, which may not have overt symptoms early on, but can lead to debilitating defects causing acute vision loss later in life. Early recognition is essential to prevent the development of complications. This presents a challenge as individuals are often asymptomatic and thus may not present for checkups until after the development of acute vision loss due to complications such as retinal detachment. Routine ophthalmology screening can help in identifying potential cases. However, no current screening programs exist for retinal detachment, and thus, it warrants further study for the development of such screening protocols. Prompt management with prophylactic laser retinopexy can reduce the development of complications and improve the quality of life of the patient.
